# Identification of loci controlling mineral element concentration in soybean seeds

**DOI:** 10.1186/s12870-020-02631-w

**Published:** 2020-09-07

**Authors:** Sidiki Malle, Malcolm Morrison, François Belzile

**Affiliations:** 1 Département de phytologie, Faculty of Agricultural and Food Sciences and Institute for Integrative and Systems Biology (IBIS), Laval University, Quebec City, Quebec, Canada; 2 Ottawa Research and Development Centre, Agriculture and Agri-Food Canada, Ottawa, Ontario Canada

**Keywords:** Soybean, Minerals, XRF, GWAS, QTL

## Abstract

**Background:**

Mineral nutrients play a crucial role in the biochemical and physiological functions of biological systems. The enhancement of seed mineral content via genetic improvement is considered as the most promising and cost-effective approach compared alternative means for meeting the dietary needs. The overall objective of this study was to perform a GWAS of mineral content (Ca, K, P and S) in seeds of a core set of 137 soybean lines that are representative of the diversity of early maturing soybeans cultivated in Canada (maturity groups 000-II).

**Results:**

This panel of 137 soybean lines was grown in five environments (in total) and the seed mineral content was measured using a portable x-ray fluorescence (XRF) spectrometer. The association analyses were carried out using three statistical models and a set of 2.2 million SNPs obtained from a combined dataset of genotyping-by-sequencing and whole-genome sequencing. Eight QTLs significantly associated with the Ca, K, P and S content were identified by at least two of the three statistical models used (in two environments) contributing each from 17 to 31% of the phenotypic variation. A strong reproducibility of the effect of seven out these eight QTLs was observed in three other environments. In total, three candidate genes were identified involved in transport and assimilation of these mineral elements.

**Conclusions:**

There have been very few GWAS studies to identify QTLs associated with the mineral element content of soybean seeds. In addition to being new, the QTLs identified in this study and candidate genes will be useful for the genetic improvement of soybean nutritional quality through marker-assisted selection. Moreover, this study also provides details on the range of phenotypic variation encountered within the Canadian soybean germplasm.

## Background

Soybean is utilized for a wide array of food, feed, and industrial purposes, making it one of the most versatile grain crops grown. In fact, soybean is an important source of protein, oils and carbohydrates, as well as other beneficial nutrients such as mineral elements which affect end-use traits of both the oil and protein fractions as well as the quality characteristics of seed used to plant succeeding crops [1]. The availability of mineral nutrients to plants is a very dynamic and complex process that is affected by both biotic and abiotic factors and their interactions [2]. In agriculture, it has been reported that deficiencies in essential elements can lead to yield loss, increased disease susceptibility, impaired metabolism, interrupted normal development and poor seed quality [3]. For this purpose, understanding the uptake, regulation, transport, and storage of mineral elements under a variety of environmental conditions is essential to deciphering the complex relationship between a plant and its environment. Therefore, the seed ionomic profiles is a powerful tool for matching a plant’s genetic characteristics with its response to environmental perturbations [4].

The enhancement of seed mineral nutrient content via genetic improvement is considered as the most promising and cost-effective approach to ensure that the dietary needs of consumers are met. As breeding for any trait rests on the existence of phenotypic variability, this requires the identification of cultivars with useful genetic variability for grain minerals and understanding the genetic architecture of these seed traits [5].

Since the concepts of plant nutrition were founded, much effort has been put into developing methods and tools for quantitative measurement of the elemental composition of living organisms [6]. Spectroscopic methods such as energy dispersive X-ray fluorescence (ED-XRF) are increasingly gaining a foothold as they are easier to operate and constitute a non-destructive tool compared to wet chemistry methods such as flame atomic absorption spectroscopy (FAAS) [7]. Recently, ED-XRF has been used successfully to assess Ca, K, P and S concentrations in soybean [8, 9], in cacao [7] and in pea seeds [10]. In these studies, measurements obtained via spectroscopy were consistent with previous studies using more common but costly analytical methods. Also, these studies have shown that mineral element content can span a large range of values in plants and that this content is determined both by genetic and environmental factors [2, 4, 11–13].

A limited number of studies have aimed to determine the genetic architecture governing the accumulation of mineral elements in seeds and concluded these traits to be most likely controlled by many genes [2, 14]. Zhang et al. [15] reported 4 QTLs (on chromosomes 7, 8 and 20) associated with calcium content in soybean seeds using 148 simple sequence repeat (SSR) markers and 178 F_2:3_ and 157 F_2:4_ lines. King et al. [13] reported 3 QTLs (on chromosomes 7, 12 and 17) for phosphorus content using 916 SSR markers and 92 F_2:4_ lines. More recently, Ramamurthy et al. [2] used 1536 single nucleotide polymorphism (SNP) markers and a total of 288 soybean recombinant inbred lines (RILs) to identify 7 QTLs associated with Ca, K and S content (on chromosomes 4, 6, 15, 16 and 18). Using a GWAS approach, Ziegler et al. [4] used 36,489 SNPs and 1653 soybean accessions from the USDA Soybean Germplasm Collection to identify 9 QTLs (on chromosomes 1, 2, 5, 9, 10 and 13) associated with Ca, K, P and S content in soybean seeds. Finally, Dhanapal et al. [16] reported a total of 65 QTLs across the 20 chromosomes associated with soybean shoot Ca, K, P and S content using 31,748 SNPs and 104 soybean genotypes. Overall, little overlap in the QTLs identified in these studies is observed. This could be due to the genetic determinants of mineral element content in soybean seeds being different among different sets of germplasm or that some studies suffered from inadequate genome coverage and failed to detect shared QTLs.

In the context of an incomplete and often inconsistent identification of QTLs controlling the accumulation of mineral elements in the soybean seed [4], especially among early maturity soybeans (MG000-II), we sought to characterize the phenotypic diversity among a set of 137 Canadian short-season soybean varieties and to identify QTLs controlling Ca, K, P and S content in this set of germplasm. Using a large set of SNP markers and three analytical approaches (CMLM, MLMM and FarmCPU), we identified a total of 32 QTLs controlling the accumulation of these four important elements of which eight were identified jointly by at least two approaches. We believe that the findings of this research will provide new insight for future research on genetic improvement of soybean seed quality and nutrient content.

## Results

### Correlation between wet chemistry and energy-dispersive X-ray fluorescence method

**Fig. 1:**
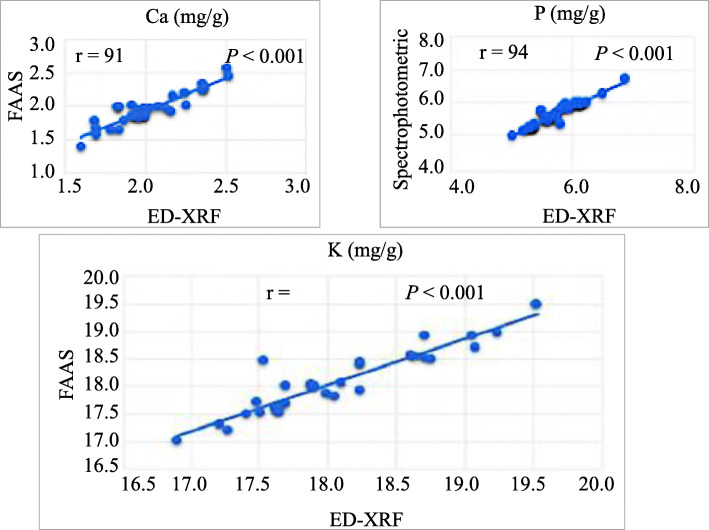
Pearson correlation between wet chemistry and ED-XRF for Ca, K, P and S content on a dry-weight basis among 30 soybean seed samples

To validate our chosen analytical method (energy-dispersive X-ray fluorescence, ED-XRF), thirty samples were analyzed by both ED-XRF and flame atomic absorption spectroscopy (FAAS) for Ca and K and by spectrophotometry for P content. As can be seen in Fig. 1, the correlation coefficients (r) between both methods were positive and highly significant (*P* <  0.001) and ranged from 0.91 (Ca) to 0.94 (P). These results demonstrated that the ED-XDF was appropriate for the quantification of Ca, P and K content in soybean seeds.

### Phenotypic variation and correlations among traits

**Fig. 2:**
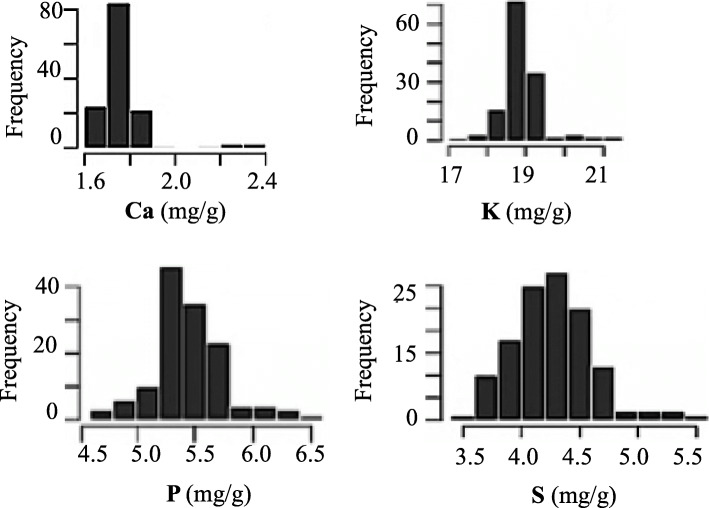
Distribution of Ca, K, P and S content in the seed of 137 Canadian soybean lines

The concentrations of Ca, K, P and S on a set of 137 soybean lines grown on two sites (two replicates/site) in 2013 were estimated using an ED-XRF device. The frequency distributions exhibited an approximately normal distribution and appeared to be quantitatively inherited (Fig. 2). As shown in Table 1, the range of seed mineral content varied for the four elements: from 1.6 to 2.4 mg/g for Ca, 17 to 21 mg/g for K, 4.5 to 6.5 mg/g for P and 3.5 to 5.5 mg/g for S content on a dry-weight basis. Across all 137 lines, the means were 1.8, 18.7, 5.3 and 4.3 mg/g respectively for Ca, K, P and S content. The least significant difference (LSD) between two genotype means was 0.03 mg/g for Ca, 0.44 mg/g for K, 0.28 mg/g for P and 0.09 mg/g for S content. A high broad-sense heritability was observed and ranged from 81% (K) to 99% (S). The presence of a fairly large phenotypic variation and high heritability suggested that these traits and association panel would be well suited to uncover the genetic architecture of these traits.

**Table 1:** Descriptive statistics for Ca, K, P and S content across two sites (two replicates per site) in the seed of 137 Canadian soybean lines

Traits	Range	Mean	LSD	H^**2**^ (%)
**Ca**	1.6–2.4	1.8	0.03	84
**K**	17.0–21.0	18.7	0.44	81
**P**	4.5–6.5	5.3	0.28	83
**S**	3.5–5.5	4.3	0.09	99

LSD = least significant difference

H2 = broad sense heritability

As illustrated in Table 2, an analysis of variance showed that both the genotype and environment had a highly significant effect (*P* ≤ 0.001) on phenotypic variation for all traits except for Ca where the genotypic effect was the sole significant source of variation. No significant genotype x environment interactions were observed for any of the traits. The observed phenotypic values were significantly (*p* <  0.001) correlated between the two experimental sites, with correlations ranging between 0.75 and 0.98. The seed content in the different minerals also proved to be correlated (Table S1, in bold). All such pairwise comparisons were statistically significant (*p* <  0.05) and the highest correlations were observed between K and S (r^2^ = 0.67, *p* <  0.001) as well as between P and K (r^2^ = 0.65, *p* <  0.001).

**Table 2:** ANOVA results for Ca, K, P and S content across two sites (two replicates per site) in seed of 137 Canadian soybean lines

Nutrient	Source of variation	df	F values	***p***-values
Ca	Genotype	136	4.69	< 0.0001***
	Environment	1	0.47	= 0.4900 **ns**
	Genotype x Environment	136	0.17	= 1.0000 **ns**
K	Genotype	136	2.72	< 0.0001***
	Environment	1	23.88	< 0.0001***
	Genotype x Environment	136	0.34	= 1.0000 **ns**
P	Genotype	136	15.32	< 0.0010 **
	Environment	1	11.01	< 0.0010 **
	Genotype x Environment	136	0.08	= 1.0000 **ns**
S	Genotype	136	19.46	< 0.0001***
	Environment	1	15.32	< 0.0001***
	Genotype x Environment	136	0.10	= 1.0000 **ns**

df = degree of freedom

*** and ** = Significant, *p* < 0.0001 and 0.001; **ns** = not significant, *p* > 0.05

### Genotyping and SNP calling

The lines of the association panel were initially genotyped via a GBS approach that yielded a total of 56 K high-quality SNPs. In a second step, a reference panel of 4.3 M SNPs was used to perform missing loci imputation onto the original set of GBS-derived SNPs. After removing InDels, markers with a MAF <  0.05 and heterozygosity > 0.1, a total of 2.18 M SNPs were retained, offering an average marker density of 1 SNP every 435 bases across the entire genome. The physical distribution of these 2.18 M SNPs across the soybean 20 chromosomes is illustrated in Fig. S1. The genotypic data thus obtained was then used to characterize population structure within this panel and to look for marker-trait associations.

### Population structure

**Fig. 3:**
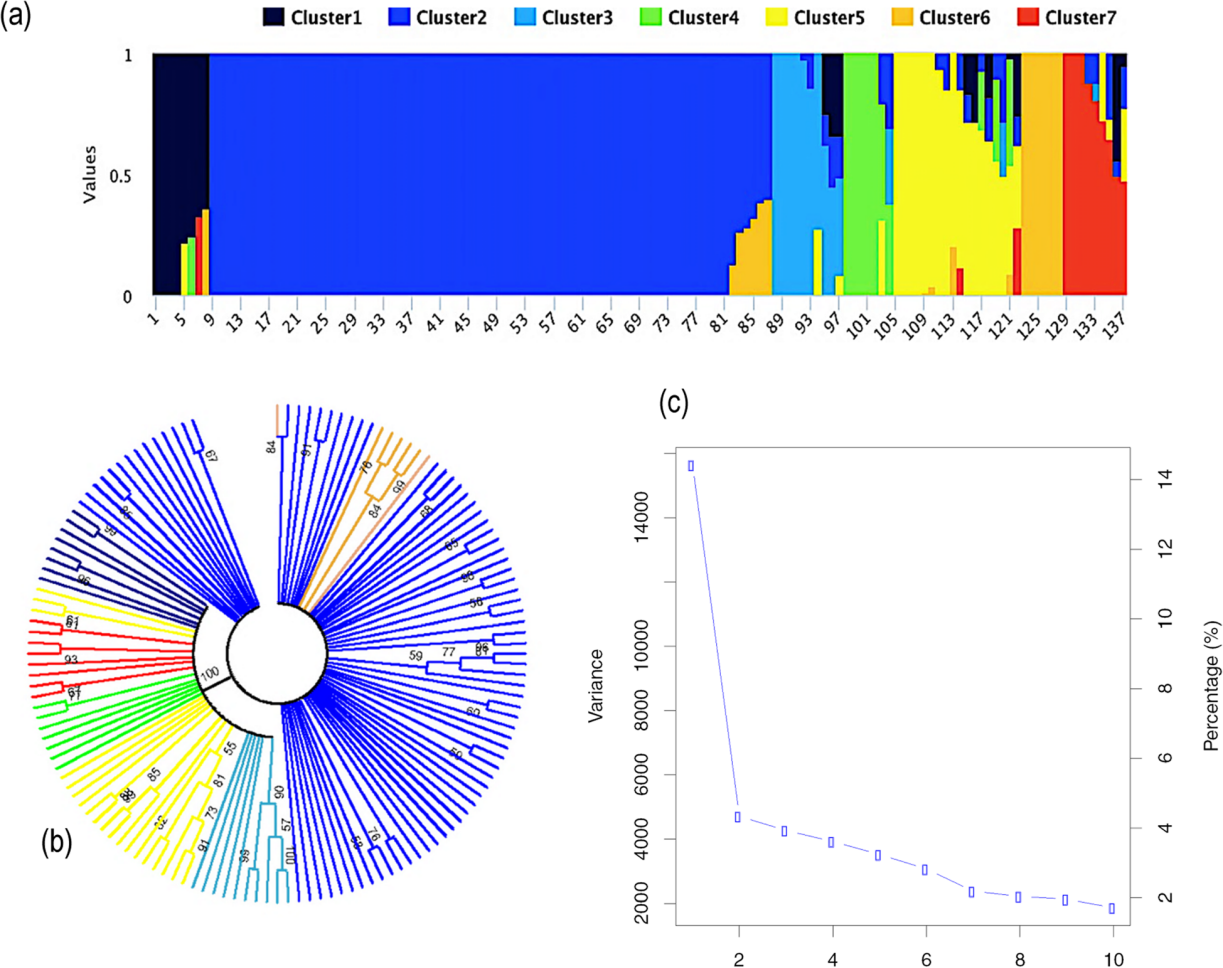
Models-based population structure in a core set of 137 Canadian soybean lines. a: Classification into seven populations using fastSTRUCTURE where each individual (from 1 to 137) is represented by a single vertical line and each color represents one cluster. b: Bootstrap consensus phylogenetic tree (2000 replicates) constructed using MEGA 7; each color represents a subgroup and seven subgroups were found in total and c: PCA eigenvalues computed using GAPIT. The total variance explained by each principal component (PC) decreased from PC1 to PC7 and, beyond PC7, the variance explained by each further PC remained low and stable

The population structure of this core set of 137 Canadian soybean lines was initially inferred using fastSTRUCTURE and the number of subpopulation (*k*) was 7 (Fig. 3a). In addition, as can be seen in Fig. 3b and c, both a phylogenetic tree and a PCA-based population structure analysis were consistent with the result of the fastSTRUCTURE analysis. Together, these results suggested that *k* = 7 provided a good assessment of population structure and the corresponding Q matrix was used for GWAS.

### Genome-wide association scan for mineral elements content in soybean seeds

To discover chromosomal regions that contribute to the phenotypic variation, we used three analytical tools to measure marker-trait associations: FarmCPU, CMLM and MLMM. As shown in the quantile-quantile (QQ) plots (Fig. S2), all three models successfully limited the confounding effects as the observed *p-*values only diverged from the diagonal (expected *p*-values) at the most extreme values (beyond 3E-03 for almost all traits).

**Fig. 4:**
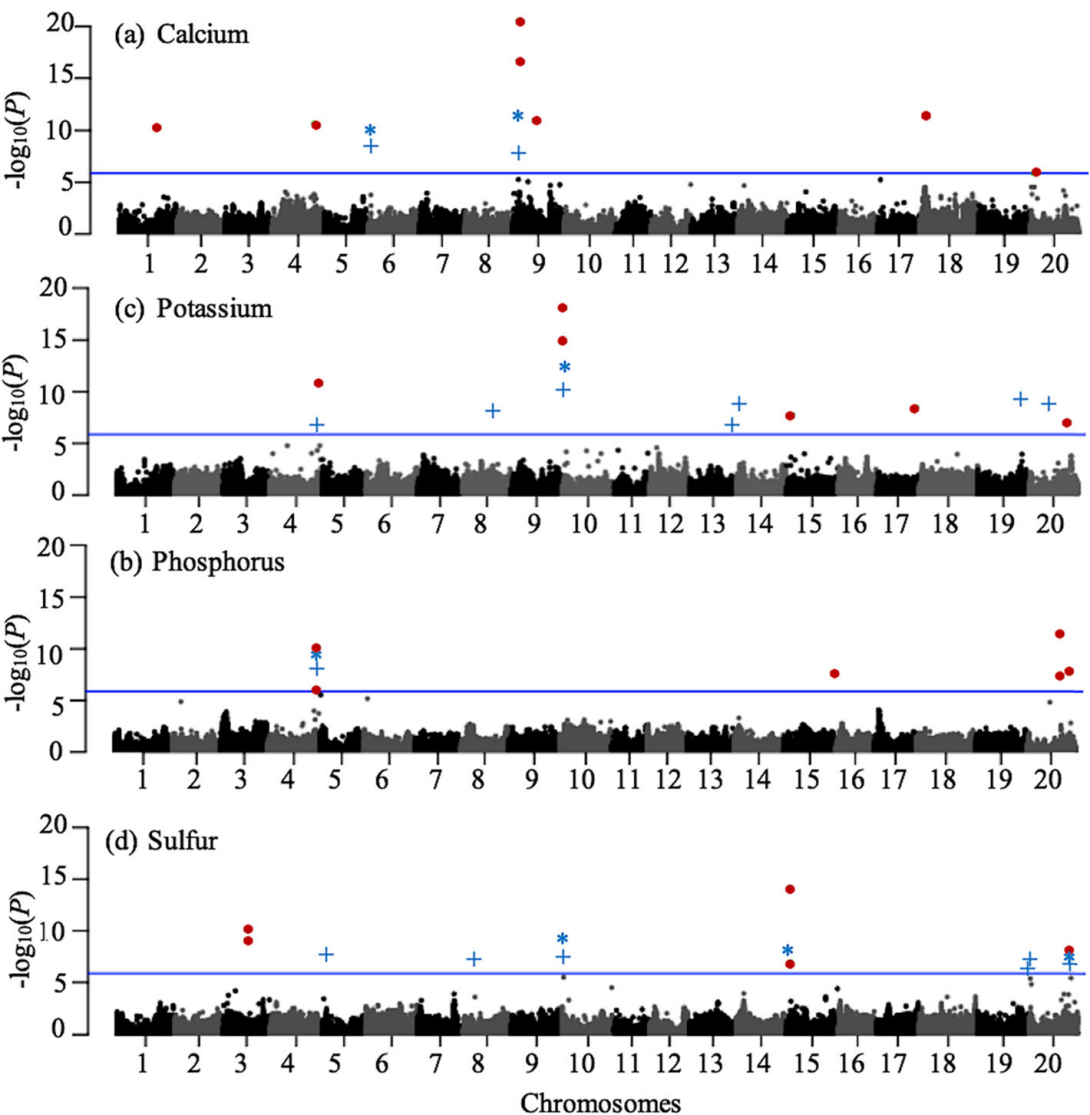
Manhattan plots for mineral elements content in a core set of 137 Canadian soybean accessions. Manhattan plots for (a) calcium (b) potassium, (c) phosphorus and (d) sulfur content. Each dot/symbol indicates the degree of association between a single marker and a trait (y-axis) while the x-axis shows the physical position of each marker. A blue horizontal line indicates the significance threshold (FDR ≤ 0.05). Significantly associated markers are indicated as a red dot for FarmCPU while the blue cross (+) and asterisk (*) indicate SNPs that were declared significantly associated by CMLM or MLMM, respectively. These associations were superimposed on the Manhattan plots produced using FarmCPU

The results of these association analyses are presented as Manhattan plots for FarmCPU, CMLM and MLMM in Fig. 4. Based on the threshold for false discovery rate (blue horizontal line, FDR ≤ 0.05), we detected 32 QTLs of which seven were associated with Ca content, ten with K, five with P and ten with S content (Table S2). Interestingly, one shared QTL contributing to both K (K_#1) and P (P_#1) was observed. The uncorrected *p-*values of these QTLs ranged from 1.35E-06 to 2.84E-21 for Ca, from 1.89E-05 to 8.05E-19 for K, from 1.17E-06 to 3.61E-12 for P and from 1.75E-05 to 6.63E-15 for S content.

**Fig. 5:**
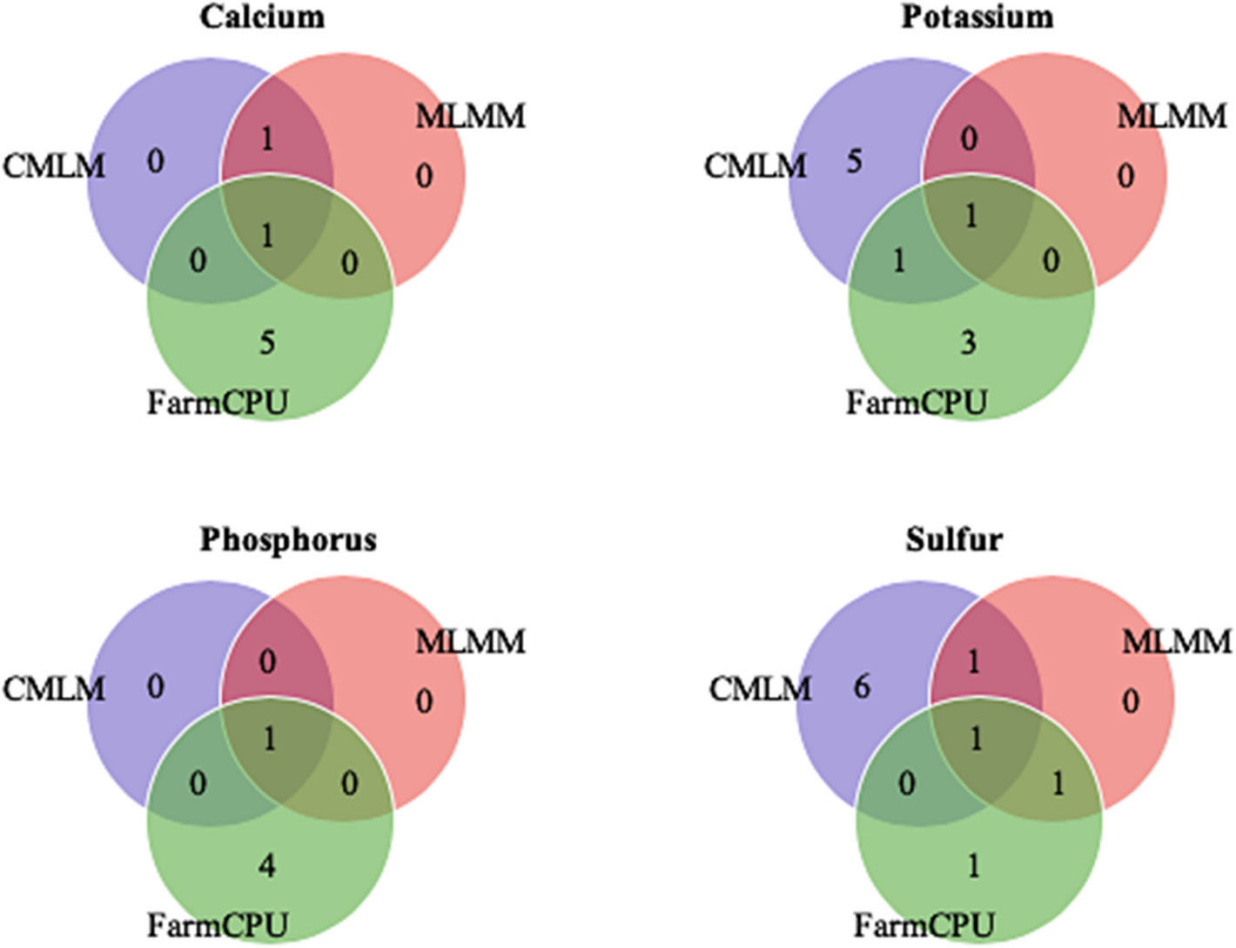
Venn diagram for the 32 identified QTLs through three analytical approaches

In total, among these 32 QTLs, eight QTLs were co-identified by at least two models (Fig. 5) and the features of these eight robust QTLs are summarized in Table 3. The portion of phenotypic variance explained (R^2^) ranged from 20 to 21% for Ca, from 17 to 31% for K, 22% for P and from 18 to 23% for S. The magnitude of allelic effects varied between 0.06 to 0.07 mg/g, 0.30 to 0.57 mg/g, 0.30 mg/g and 0.15 to 0.46 mg/g for Ca, K, P and S, respectively. The genetic variance (additive) explained as the narrow-sense heritability (h^2^) was 41% for Ca, 82% for K, 78% for P and 93% for S.

**Table 3:** List of QTLs for mineral element content identified by at least two approaches in 137 Canadian soybean lines. The most highly associated SNP within each QTL is indicated along with the associated statistics. For each trait, a measure of its heritability (h^2^) is provided. The models that detected a significant marker-trait association are abbreviated as follows: C for CMLM, M for MLMM and F for FarmCPU

Gm	Peak SNP	QTL N°	***p***-value	FDR	R2%	Effect	h^**2**^%	Models
06	3,354,869	Ca_#3	2.94E-08	4.5E-03	20	−0.06	41	C/M
09	6,092,970	Ca_#4	3.70E-08	4.5E-03	21	−0.07		C/M/F
04	49,071,552	K_#1	1.75E-06	6.1E-03	17	−0.30	82	C/F
10	1,925,709	K_#3	4.31E-10	4.9E-05	31	−0.57		C/M/F
04	49,071,286	P_#1	6.12E-08	1.5E-02	22	−0.30	78	C/M/F
10	1,602,998	S_#4	2.84E-08	4.0E-03	23	0.46	93	C/M
15	3,986,243	S_#7	2.80E-07	2.3E-02	19	0.15		M/F
20	39,076,484	S_#10	9.13E-07	9.7E-03	18	0.20		C/M/F

FDR = False discovery rate

R2% = Indicates the proportion of total phenotypic variation for each marker

### Validation of the eight co-identified QTL across three environments

**Fig. 6:**
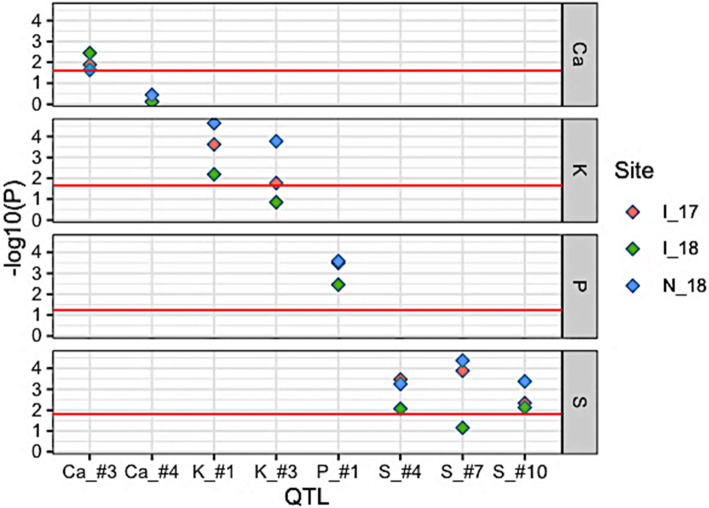
Stability of the eight QTLs detected by at least two models for Ca, K, P and S content. The core set of 137 early Canadian soybean accessions were grown in three additional environments (in 2017 or 2018, with [I_] or without [N_] supplemental irrigation). The phenotypic mean was calculated for the subsets of lines contrasting for the peak SNP at each of 8 QTLs previously detected by at least two of the three GWAS models. Each colored symbol represents the *p*-value for the contrast observed in one environment. The y-axis shows the -log10(*p*-value) of each test while the x-axis shows the reported QTLs associated with each trait. A red horizontal line indicates the Bonferroni significance threshold at ≤ − log10 (0.05/n), where n = number of co-identified QTLs per trait (e.g. 0.05/2 for Ca)

To verify the stability of each of the eight QTLs detected by at least two models, data from three additional trials were obtained. Overall, across the three new environments, seven QTLs were validated in at least two environments (Fig. S3 and Table S3). Only QTL#4 for Ca could not be validated in any of the three new environments. The I_18 environment saw the lowest rate of validation with five QTLs being successfully detected in this environment (Fig. 6). Of the 24 possible QTL-environment combinations (8 QTLs × 3 environments), 18 resulted in a significant difference between the mean phenotype of lines contrasting for the peak SNP. These results indicate that the identified QTLs are robust across a wide range of environments.

### Refinement of the GWA scan for co-identified QTL

To more deeply explore variants in these robust QTLs, we extracted all SNPs falling within the haplotype blocks surrounding the seven most robust QTLs from the larger catalogue of 2.2 M SNPs. These were merged with the pruned data (243 K) set to perform the GWAS with three models again. In six of these seven instances, stronger association signals were observed and the physical distance between the previous and the new peak SNP ranged from 1 to 311 kb (Table S4), but always resided within the same haplotype block.

### Prediction of candidate genes within the robust QTL regions

**Fig. 7:**
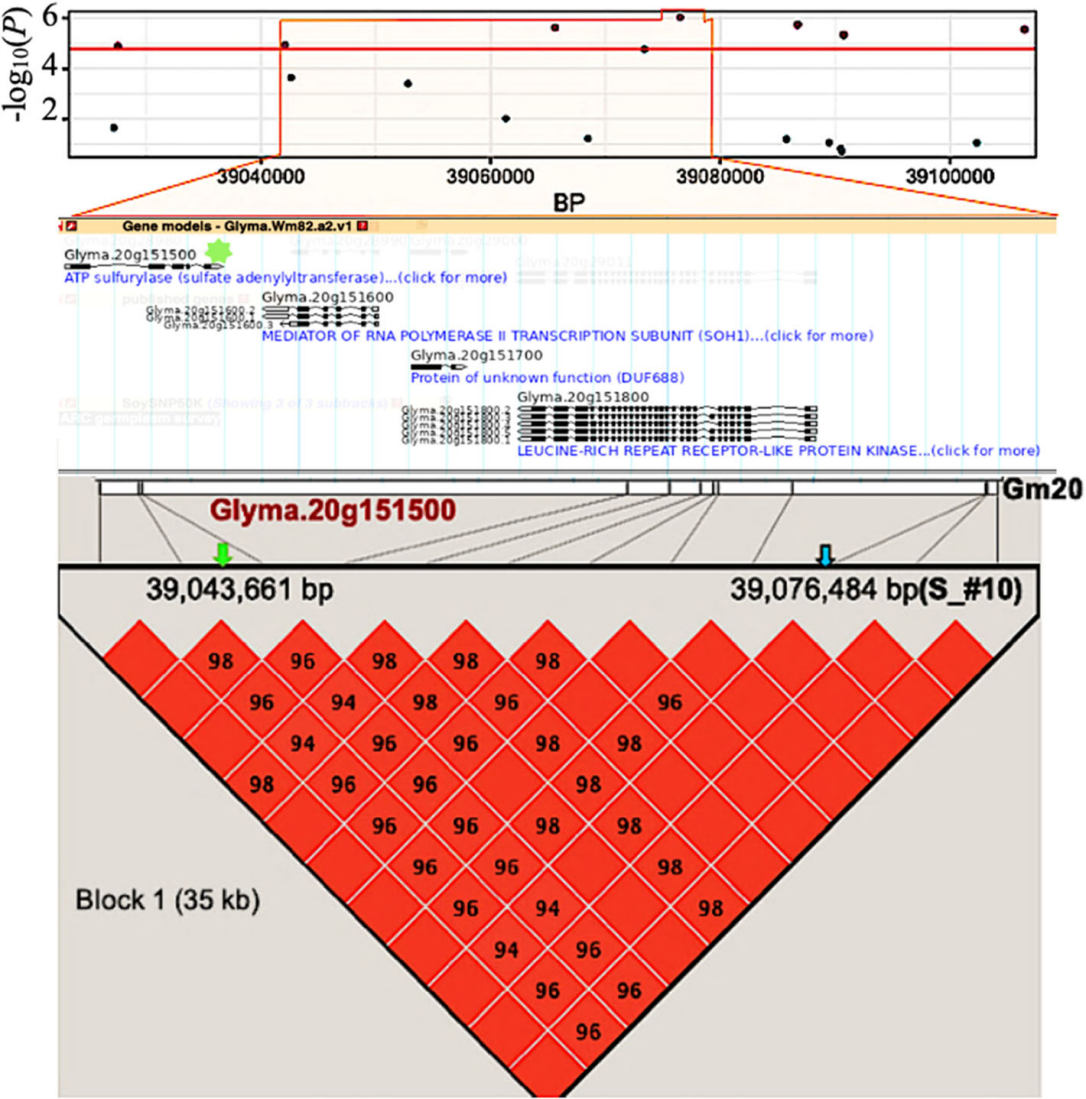
Identification of a candidate gene underlying QTL S_#10 within the haplotype block on chromosome 20. Top panel: marker-trait associations within a ~ 80-kb interval (39,027–39,106 Kb) of Gm20. Middle panel: position and orientation of four gene models present in the 35-kb region that is defined by the left-most (Gm20: 39,042,071) and right-most (Gm20: 39,076,880) markers that are in perfect LD with the peak SNP (Gm20:39,076,484). The most likely candidate gene (Glyma.20G151500, Sulfate assimilation) is highlighted with a green asterisk. Bottom panel: pairwise LD among markers falling within the defined genomic region of interest. LD is indicated as D’× 100 and the empty squares indicate complete LD (D’ = 1). The position of the peak SNP (blue arrow) and candidate gene (green arrow) are shown

Based on the GWAS results, we investigated the genes annotated in the soybean genome in order to identify putative candidate genes from loci significantly associated with each trait. To establish a list of candidate genes, we focused only on those residing within a region delimited by the left-most and right-most flanking markers that were in perfect LD (D’ = 1) with the peak SNP for the seven QTLs described above. These genomic regions (ranging in size between 32 and 360 kb) were extracted from Wm82.a2.v1 and the GO annotations of genes residing within these regions was examined (Table 4). An example of this approach is illustrated in Fig. 7. The number of genes residing (fully or in part) in each region varied between 4 and 43 and the full list of these genes and their annotations are provided in Table S5.

**Table 4:** Identification of candidate genes for seven QTLs associated with mineral element content in a core set of 137 Canadian soybean lines. For each robust QTL (detected using multiple models in many environments), a region of interest was delimited by flanking markers in perfect LD with the peak SNP. The identifier and annotation of candidate genes residing within the relevant genomic regions are provided

Gm	QTL	Peak SNP	Size of LD block	# of genes	Candidate gene	Relevant annotation
06	Ca_#3	3,354,869	199 kb	30	Glyma.06G046000	Calcium ion transport
04	K_#1	49,071,552	32 kb	4	NA	NA
10	K_#3	1,966,469	360 kb	43	Glyma.10G020000	Potassium ion transport
04	P_#1	49,071,286	32 kb	4	NA	NA
10	S_#4	1,602,998	162 kb	18	NA	NA
15	S_#7	3,986,243	158 kb	20	NA	NA
20	S_#10	39,076,484	35 kb	04	Glyma.20G151500	Sulfate assimilation

To identify a candidate gene, we looked for genes that met either of the two following criteria: 1) genes annotated as being involved in the transport of the given mineral element and expressed in roots, shoots or leaves or 2) genes annotated as being involved in the uptake, translocation, and/or homoeostasis of the element of interest and mainly expressed in seeds. In total, three promising candidate genes involved either in the transport or assimilation of these mineral elements were identified. We first discovered Glyma.06G046000 (132 kb upstream of the peak SNP in Ca_#3), Glyma.10G020000 (222 kb downstream of the peak SNP in K_#3). These two genes were both annotated as being involved in transport and expressed in roots tips and roots hairs. In addition, Glyma.06G046000 was expressed in young leaves, flowers, main roots, pods as well as in seeds (Fig. S4b and 4d.). Finally, Glyma.20G151500 (32 kb downstream of S_#10) was annotated as being involved in sulfate assimilation and expressed in flowers, roots, nodules and seeds (Fig. S4f). No candidate gene falling within the defined LD blocks and meeting our criteria was found for QTLs K_#1, P_#1, S_#4 and S_#7.

### Structural and nucleotide variation within candidate genes and their predicted functional impact

To determine if genetic (structural or nucleotide) variation within or overlapping the candidate gene could constitute causal variants, we examined a catalogue of such variation established from the whole-genome sequencing data available for a subset of 56 lines. No structural variant (> 51 bp) was identified as overlapping in full or in part with these three candidate genes. As for nucleotide variants, a total of 18 SNPs were found within the coding regions of two genes (one within Glyma.06G046000 and 17 within Glyma.20G151500). All of these variants were predicted as a having a “modifier” or “low” impact on protein function. It is therefore unlikely that the observed phenotypic variation is due to a loss of function of these candidate genes.

To provide more insight into the involvement of these candidate genes in the observed phenotypic variation, a haplotype analysis was performed. As presented in Fig. S5, significant phenotypic differences (*P* <  0.05) were observed between the haplotypes identified for each candidate gene. For example, among the four haplotypes (A, B, C and D) identified for Glyma.06G046000, the seven lines carrying haplotype B exhibited a significantly different Ca content compared to the 130 other lines carrying haplotypes A, C or D. Similarly, a small group of five accessions carrying haplotype C at Glyma.10G020000 had a higher K content than the other accessions carrying one of the four other haplotypes. In the case of Glyma.20G151500, it was the more frequent haplotype A (*n* = 99) that exhibited a significantly higher S content than the other four haplotypes. These results support that each candidate gene is highly promising as they each contributed to the phenotypic variation.

## Discussion

### Phenotypic variation and correlations among traits

Across the two environments used to perform the original discovery of marker-trait associations, the seed contents for all the elements (Ca, K, P and S) were normally distributed and suggested that they are quantitatively inherited. The phenotypic variation in this study ranged from 1.6 to 2.4 mg/g for Ca content, 17 to 21 mg/g for K content, 4.5 to 6.5 mg/g for P content and 3.5 to 5.5 mg/g for S content on a dry-weight basis. In previous studies, different ranges have been reported. Otaka et al. [8] and Homura et al. [17] reported similar seed content for Ca (1.5 to 3.5 mg/g and 1.5 to 3.2 mg/g, respectively) and K (19.8 to 22.1 mg/g and 16.7 to 21.2 mg/g, respectively). Similarly, King et al. [13] reported a comparable range of values for P content (3.9 to 5.6 mg/g), while Dhanapal et al. [16] reported a lower range of values (1.3 to 4.9 mg/g). However, the range of S content in this study was slightly higher than what has been previously reported. Our values for S content were generally higher than those reported by Fageria [18] and Bellaloui et al. [19] (2.5 to 4.0 mg/g and 1.6 to 3.1 mg/g, respectively). Overall, the values reported here were fairly consistent with those reported in previous studies and the slight differences in range may simply reflect differences due the specific set of accessions grown in different environments as well as the choice of measurement method. Nonetheless, in the context of an association study, the accuracy of the phenotypic values is not as important as adequately capturing how the values vary across the panel.

In our study, the broad-sense heritability estimated across the two environments was high (H^2^ > 0.80) for all traits. Such relatively high broad-sense heritability suggested that the phenotype was largely determined by the genotypic effect [20]. Similar heritabilities for these traits have been reported in previous studies (H^2^ = 0.48 to 0.93) [14, 16]. In addition, a significant and positive correlation between the concentrations of K and P was observed (0.65, Table S1). This is consistent with previous reported results which ranged from 0.80 to 0.94 [2, 19] .

### Genome-wide association scan for mineral elements content in soybean seeds

A total of 32 QTLs associated with the Ca, K, P and S content were identified (Table S2) and eight of these were detected by at least two models. More importantly, seven of these eight QTLs proved extremely robust as they could be successfully confirmed as impacting mineral element content in three additional trials. In previous GWAS studies, Ziegler et al. [4] and Ning et al. [14] reported 22 and 9 QTLs, respectively. Using a linkage mapping approach, Ramamurthy et al. [2] reported 7 and Bellaloui et al. [19] reported 11 QTLs associated with soybean seed Ca, K, P and S content. The large initial number of QTLs detected in this work (32) can potentially be ascribed to a more exhaustive genome coverage (2.2 M SNPs) and to the use of multiple models for detecting marker-trait associations. It is unlikely to be due to a particularly wide range of phenotypic values in our association panel as this range was comparable to those reported in previous studies.

The QTLs described in this work generally explained a fairly substantial portion of the phenotypic variance (18–31%). The phenotypic variance explained by previously reported QTLs varied from 2 to 18%. Interestingly, one of our QTLs was found to impact both K and P content. This shared QTL for K and P content is not surprising given the high degree of positive correlation between these two elements. Similarly, Dhanapal et al. [16] also reported a QTL associated with the content in these two elements. Such correlations could be due to shared physiological mechanisms and metabolic pathways [4, 21]. In other words, it may have occurred either by pleiotropy of the same gene involved in controlling these mineral concentrations such as a co-transporter [20] or simply by the presence of independent genes in the same regions.

In order to compare our results with previously identified QTL regions, we queried the previous QTLs against the SoyBase genome browser and defined their physical position. None of the seven robust QTLs identified in this study coincided with previously reported QTL intervals identified either in family-based mapping or GWAS. Thus, the current QTLs can be considered novel. This absence of overlap between the QTLs identified through this work and those reported previously may reflect the fact that these traits are determined by different genes in the experimental materials used in the different mapping experiments.

### Candidates genes and their functions for mineral elements accumulation

As mentioned above, we focused our attention on transport-related genes that were also expressed in roots, shoots or leaves and genes annotated to be involved in nutrient uptake, translocation, and/or homoeostasis mainly expressed in seeds. For K content, a transport-related gene (Glyma.10G020000) was identified underlying QTL K_#3 on Gm 10. This gene was annotated as a K^+^ potassium transporter and its ortholog in *A. thaliana* (*AT4G13420.1*) encodes a high affinity K^+^ transporter 5 (HAK5). In rice, Yang et al. [22] demonstrated that HAK5 plays important roles in controlling both the influx of K^+^ into roots and its transport to the aerial parts of the plant. Two paralogs of our candidate gene (Glyma.02 g154100 and Glyma.07 g042500) were functionally characterized as being involved in the root uptake of K^+^ in soybean [23, 24]. Interestingly, in the work of Dhanapal et al. [16], QTLs for K content do overlap with the genomic positions of these HAK5 paralogs. The fact that we did not detect any association between these paralogs and K content in our study suggests that it is variation in the HAK5 paralog on chromosome 10 (Glyma.10G020000) that contributes to differential accumulation of K in Canadian early-maturing soybean lines.

For S content, we identified a gene (Glyma.20G151500) that codes for ATP sulfurylase 1, the first enzyme known to be involved in the sulfate assimilation pathway in *A. thaliana (AT3G22890.1; ASA1*) [25]. An *A thaliana* cDNA encoding *ASA1* successfully complemented a *Saccharomyces cerevisiae* ATP sulfurylase mutant (*met3*), thereby restoring both methionine heterotrophy and sulfate transport [26]. Intriguingly, a paralog of our candidate gene was found on Gm10 by [16] (Glyma.10 g242600). This again suggests that the same enzymatic activity is contributing to S accumulation, but that different copies of the gene control S content in different sets of germplasm.

Finally, for Ca content, the candidate gene Glyma.06G046000 was annotated as a calcium transporting ATPase involved in calcium transport. This gene is orthologous to an *A. thaliana* locus (*AT1G27770.1*; ACA1) that encodes a chloroplast envelope Ca2^+^-ATPase which is known to bind the calmodulin that leads to activation of a Ca2^+^ pump [27]. It has been shown that Ca2^+^-ATPases are enzymes that actively transport Ca2+ in eukaryotic cells [28] and are involved in all stages of the plant life cycle including growth and development [29].

## Conclusions

Compared to previous studies, the high density of markers used in this study has contributed to the reproducible detection of several new loci associated with the content of mineral elements in soybean seeds. In addition to providing details on the range of phenotypic variation encountered within the Canadian soybean germplasm for mineral elements content in the seeds, this study also provided more information on the genetic architecture underlying their accumulation. The markers and genes identified in this study will be useful for the genetic improvement of soybeans through marker-assisted selection.

## Methods

### Plant material and experimental design

A set of 137 of early maturing soybean lines (belonging to maturity groups 000-II, MG000-II), was selected from a larger group of 304 accessions based on the analysis of population structure as described in Sonah et al [30] to be representative of the genetic diversity in Canadian short-season soybean. Soybean lines were sourced from Drs. Louise O’Donoughue (CÉROM, St-Mathieu-de-Beloeil, QC), Elroy Cober (Agriculture and Agri-Food Canada, Ottawa, ON), Istvan Rajcan (University of Guelph, Guelph, ON) and Mr. Éric Gagnon (Semences Prograin Inc., St-Césaire, QC). In a first (discovery) phase, lines were phenotyped in two environments, namely Woodstock (ON) and St-Mathieu-de-Beloeil (QC) in Canada in 2013. The experimental design was a generalized lattice in which all lines were planted in a single-row plot with two replicates at each location. In a second (validation) phase, the same lines were grown in three environments at the Central Experimental Farm in Ottawa (ON) in 2017 (17) and 2018 (18). The lines were planted in a modified augmented design as four-row plots with a single replicate. Within each year, two different treatments were applied: no irrigation (N) or drip irrigation (I). As a full set of lines/seed was not available for the N_17 trial, the robustness of the discovered QTLs was carried out using data from I_17, I_18 and N_18 trials only.

### Calibration and validation

Calibration of the energy-dispersive X-ray fluorescence (ED-XRF) spectrometer was achieved by an empirical calibration approach [31] in which sets of standards with similar composition and morphology to the samples of interest were used. Here, elemental concentrations were measured in a set of samples using flame atomic absorption spectrometry (FAAS) for Ca and K as per [32] and by spectrophotometry for P content as per [33]. For S content, we proceeded by successive addition of Na_2_SO_4_ on reference materials (WEPAL, IPE 885 (Maize)) supplied by the National Institute of Standards and Technology (NIST) whose S content was known. The values thus obtained served as baselines to calibrate the standard curve of our ED-XRF device (Niton XL3t955 GOLDD). To determine the accuracy and reliability of our ED-XRF measurements, thirty seed samples were analyzed by both ED-XRF and wet chemistry for their concentration in Ca, K and P. no wet chemistry validation was necessary for S content.

### Phenotyping and statistical analysis

For each sample, 10 g of whole seeds were ground using a grinder (Foss A/S: Cyclotec™ 1093 Sample Mill). A 0.3-g sample of homogenous fine powder from each line was pressed using a stainless-steel pellet die in a hydraulic pellet press (Carver 4350.L) to produce compact 13-mm pellets (~ 0.2 mm thick). The pellets were stored until the measurement of Ca, K, P and S content by the ED-XRF.

Descriptive statistics, genotypic variance, environment and genotype by environment effects as well as correlation analysis between these mineral contents were performed using an R package « lmer ». To combine information from different environments, best linear unbiased predictions (BLUPs) were calculated using the restricted maximum likelihood in META-R [34]. The broad-sense heritability *H*^2^ across environments was calculated as follows:

$$ {\mathrm{H}}^2=\frac{\upsigma_{\mathrm{g}}^2}{\upsigma_{\mathrm{g}}^2+{\upsigma}_{\mathrm{g}\mathrm{e}}^2/\mathrm{nEnv}+{\upsigma}_{\mathrm{e}}^2/\left(\mathrm{nEnv}\ \mathrm{x}\ \mathrm{nrep}\right)\ } $$

where ${\upsigma}_{\mathrm{g}}^2,{\upsigma}_{\mathrm{g}\mathrm{e}}^2$ and ${\upsigma}_{\mathrm{e}}^2$ are the genotype, the genotype × environment interaction and the error variance component, respectively. The nEnv is the number of environments, and nrep is the number of replicates.

### Genotyping and SNPs imputation

A total of ~ 203 million 100-bp Illumina HiSeq2000 single-end reads derived from sequencing 192-plex GBS libraries were available for the 137 lines (as detailed previously [30]). Briefly, the restriction enzyme *Ape*K1 was used to produce a single 192-plex GBS library (containing additional unrelated samples) that was sequenced on a single lane of an Illumina HiSeq2000 sequencer. Approximately 203 million 100-bp single-end reads were obtained for the entire population of 137 lines. The Fast-GBS pipeline [35] and the Wm82.a2.v1 reference genome [36] were used for SNP calling with a minimal read depth of two reads and removing loci with more than 80% missing data. A first imputation step of missing genotypes was performed on this set of GBS-derived SNPs using BEAGLE v5 [37]. Secondly, a reference panel of 4.3 M SNPs, obtained from whole-genome resequencing of a set of 102 partially overlapping (56 shared) lines was used to perform missing loci imputation on the set of GBS-derived SNPs [38]. The accuracy of imputation of such untyped loci was previously assessed [38] and found to be 96.4%.

### Population structure and kinship analyses

For the population structure analysis, we used a pruned (r^2^ > 0.5) catalogue of 14 K SNPs obtained using Plink v1.9 [39]. The Bayesian model implemented in the program fastSTRUCTURE [40] was used to analyze the population stratification. The number of subpopulations (*k*) was set from 1 to 12 with 3 independent itterations. The number of subpopulations (*k*) was determined using a python script (“choosek”) implemented in fastSTRUCTURE. In addition to fastSTRUCTURE, two different methods were used to infer population structure: (i) a consensus phylogenetic tree computed with the Tamura-Nei model with a boostrapping based on 2000 iterations, implemented in MEGA7 [41] and (ii) a principal component analysis (PCA) implemented in the program GAPIT [42].

### Genome-wide association analysis

Genome-wide association between markers and the phenotypes was assessed in GAPIT using a pruned catalogue of 243 K SNPs (r^2^ > 0.9) and the BLUP values for each trait. CMLM was used for single-locus GWAS while MLMM and FarmCPU were used for multi-locus GWAS. The genetic relatedness between the lines conveyed through the kinship matrix (K) and the population structure matrix (Q) estimated through fastSTRUCTURE were used to control for false positive associations. The threshold of significance of marker-trait associations for the three models (CMLM, MLMM and FarmCPU) was an adjusted *p*-value with the false discovery rate (FDR) set at ≤0.05, as per the Benjamini and Hochberg procedure [43]. We assumed that all significant marker-trait associations marked the same QTL when these markers resided within the same haplotype block (a region delimited by the left-most and right-most flanking markers that were in perfect LD (D’ = 1) with the peak SNP). When different peak SNPs were detected by the different models, the one detected by two models was chosen. In addition, when the same peak SNP was detected by at least two models, the lowest uncorrected *p*-value was reported. We chose to report and investigate only co-detected QTLs, ones that were detected by at least two models for each trait. For the identification of candidate genes, genomic regions of interest surrounding a peak SNP were defined as extending between the left-most and right-most flanking markers that were in perfect LD (D’ = 1) with the peak SNP.

### Validation of the allelic effect of the co-detected QTLs in three environments

To assess the reproducibility of the QTLs identified in the discovery phase, we validated the allelic effects of the QTLs in three different environments (I_17, I_18 and N_18) using the same set of 137 lines. Marker-trait associations were tested using a t-test. The population was divided into two groups according to the allelic class at the peak SNP. We performed a t-test between the mean phenotypic values of the two groups. The threshold of significance for marker-trait associations was adjusted for multiple tests (α = 0.05/n, where n is the total number of QTLs for a trait). The significance test was assessed using a t-test function implemented in R version 3.5.1 according to the equation described in [44]: Y = μ + f (marker) + error, where Y is equal to the trait value, μ is equal to the population mean, and f (marker) is a function of the significant markers.

### Candidate genes and their functional analysis

By using a data mining algorithm [45], all genes residing within haplotype blocks of interest were extracted from the SoyBase Browser, and their GO annotations were examined. After identifying a candidate gene, further analyses were performed to identify in what tissues and at which developmental stages these candidate genes were expressed using the electronic fluorescent pictograph (eFP) Browser (www.bar.utoronto.ca) for soybean.

Altered transcripts resulting from potential loss-of-function (LOF) alleles among the list of candidate genes were investigated by inspecting the catalogue of structural variants reported by [46]. For LOF analysis, only the whole-genome sequencing dataset (56 lines) was used. For the study nucleotide variants located within genic regions, SnpEff [47] was used with the full set of nucleotide variants (prior to pruning, 2.18 M SNPs).

To further support the involvement of candidate genes in the observed phenotypic variation, a gene-centric haplotype (GCH) approach was used to identify polymorphic markers that defined the haplotypes inside the candidate genes using HaplotypeMiner [48] and the full catalogue of 2.18 SNPs. A t-test was then used between the mean phenotypic values for each haplotype.

## Supplementary information

**Additional file 1.** Table S1: Phenotypic correlation between the seeds minerals content. Table S2: The 32 reported QTLs associated with Ca, K, P and S content in seeds. Table S3: The t-test results. Table S4: Summary of localized GWAS results. Table S5: The full list of all genes residing in the significant haplotype blocks.

**Additional file 2 **Fig. S1: Distribution of the 2.18 M SNPs in the soybean genome. Fig. S2: Quantile-quantile (Q-Q) plot of *p*-values from the association study. Fig. S3: The boxplot and p-values for t-test result. Fig. S4: Identification of candidate genes underlying QTLs. Fig. S5: Candidate gene haplotypes and their phenotypic contrast.

## Data Availability

The datasets used and analyzed during the current study are available from the corresponding author upon reasonable request.

## Abbreviations

BLUP: Best linear unbiased predictor
CMLM: Compressed mixed linear model
FarmCPU: Fixed and random model circulating probability unification
GWAS: Genome-wide association study
H ^2^: Broad-sense heritability
h ^2^: Narrow-sense heritability
LD: Linkage disequilibrium
LOF: Loss of function
MAF: Minor allele frequency
MLMM: Multi-locus mixed linear model
QTL: Quantitative trait loci
R ^2^: Phenotypic variance explained
SNP: Single nucleotide polymorphism
