# Supplementary material for: Identification of loci controlling mineral element concentration in soybean seeds
Source: BMC Plant Biol. 2020 Sep 7;20:419. doi: 10.1186/s12870-020-02631-w (PMC7487956; doi:10.1186/s12870-020-02631-w)
Supplement: Supplementary file 1 — Additional file 1. Table S1: Phenotypic correlation between the seeds minerals content. Table S2: The 32 reported QTLs associated with Ca, K, P and S content in seeds. Table S3: The t-test results. Table S4: Summary of localized GWAS results. Table S5: The full list of all genes residing in the significant haplotype blocks. [file 12870_2020_2631_MOESM1_ESM.docx]

**Table S1:** Phenotypic correlation between the seeds minerals content.

| Correlation between the two experimental sites | | | | |  | Correlation between different minerals | | | |
| --- | --- | --- | --- | --- | --- | --- | --- | --- | --- |
|  | Ca | K | S | P |  | **Traits** | **Ca** | **K** | **S** |
| Ca | 0.92*** |  |  |  |  | **K** | 0.20* |  |  |
| K |  | 0.80*** |  |  |  | **S** | 0.21* | 0.67*** |  |
| S |  |  | 0.98*** |  |  | **P** | 0.18* | 0.65*** | 0.47*** |
| P |  |  |  | 0.75*** |  |  |  |  |  |

* Significant at the 0.05 probability level.

*** Significant at the 0.001 probability level.

**Table S2:** The 32 reported QTLs associated with Ca, K, P and S content in seeds. The models that detected a significant marker-trait association are abbreviated as follows: C for CMLM, M for MLMM and F for FarmCPU.

| **Traits** | **Gm** | **Peak SNP** | **QTL N°** | **P** | **R^2^** | **FDR** | **Effect** | **MODELS** |
| --- | --- | --- | --- | --- | --- | --- | --- | --- |
| Ca | 1 | 37,012,421 | Ca_#1 | 6.02^E^-11 | NA | 2.5^E^-06 | 0.05 | F |
| Ca | 4 | 41,894,618 | Ca_#2 | 2.12^E^-11 | NA | 1.0^E^-06 | -0.05 | F |
| Ca | 6 | 3,354,869 | Ca_#3 | 2.94^E^-08 | 0.20 | 4.5^E^-03 | -0.06 | C/M |
| Ca | 9 | 6,092,970 | Ca_#4 | 3.70^E^-08 | 0.21 | 4.5^E^-03 | -0.07 | C/M/F |
| Ca | 9 | 21,562,118 | Ca_#5 | 1.05^E^-11 | NA | 6.4^E^-07 | 0.01 | F |
| Ca | 18 | 4,907,739 | Ca_#6 | 4.11^E^-12 | NA | 3.3^E^-07 | -0.03 | F |
| Ca | 20 | 3,830,121 | Ca_#7 | 1.35^E^-06 | NA | 4.7^E^-02 | -0.02 | F |
|  |  |  |  |  |  |  |  |  |
| K | 4 | 49,071,552 | K_#1 | 1.75^E^-06 | 0.17 | 6.1^E^-03 | -0,30 | C/F |
| K | 8 | 47,147,391 | K_#2 | 4.22^E^-07 | 0.19 | 2.0^E^-03 | -0,41 | C |
| K | 10 | 1,925,709 | K_#3 | 4.31^E^-10 | 0.31 | 4.9^E^-05 | -0,57 | C/M/F |
| K | 14 | 1,771,702 | K_#4 | 4.37^E^-08 | 0.23 | 3.8^E^-04 | -0,52 | C |
| K | 14 | 42,498 | K_#5 | 3.21^E^-06 | 0.16 | 9.3^E^-03 | -0,27 | C |
| K | 15 | 6,097,078 | K_#6 | 1.78^E^-08 | NA | 8.7^E^-04 | 0,17 | F |
| K | 17 | 39,070,739 | K_#7 | 3.54^E^-09 | NA | 2.2^E^-04 | 0,20 | F |
| K | 19 | 49,929,548 | K_#8 | 7.20^E^-09 | 0.26 | 1.2^E^-04 | -0,48 | C |
| K | 20 | 31,865,721 | K_#9 | 1.54^E^-08 | 0.24 | 2.1^E^-04 | -0,48 | C |
| K | 20 | 39,090,716 | K_#10 | 9.01^E^-08 | NA | 3.7^E^-03 | 0,13 | F |
|  |  |  |  |  |  |  |  |  |
| P | 4 | 49071286 | P_#1 | 6.12^E^-08 | 0.22 | 1.5^E^-02 | -0.30 | C/M/F |
| P | 15 | 48783732 | P_#2 | 2.16^E^-08 | NA | 1.3^E^-03 | 0.13 | F |
| P | 20 | 31872237 | P_#3 | 3.61^E^-12 | NA | 8.8^E^-07 | -0.09 | F |
| P | 20 | 33742119 | P_#4 | 3.58^E^-08 | NA | 1.7^E^-03 | -0.06 | F |
| P | 20 | 42478388 | P_#5 | 1.35^E^-08 | NA | 1.1^E^-03 | 0.09 | F |
|  |  |  |  |  |  |  |  |  |
| S | 3 | 22,917,408 | S_#1 | 7.81^E^-11 | NA | 9.5^E^-06 | 0.10 | F |
| S | 5 | 1,095,389 | S_#2 | 1.17^E^-05 | 0.14 | 4.0^E^-02 | 0.26 | C |
| S | 8 | 12,150,272 | S_#3 | 4.93^E^-06 | 0.15 | 2.1^E^-02 | 0.30 | C |
| S | 10 | 1,602,998 | S_#4 | 2.84^E^-08 | 0.23 | 4.0^E^-03 | 0.46 | C/M |
| S | 14 | 58,133 | S_#5 | 2.69^E^-07 | 0.19 | 6.6^E^-03 | 0.26 | C |
| S | 14 | 1,755,083 | S_#6 | 5.32^E^-07 | 0.18 | 8.1^E^-03 | 0.43 | C |
| S | 15 | 3,986,243 | S_#7 | 2.80^E^-07 | 0.19 | 2.3^E^-02 | 0.15 | M/F |
| S | 19 | 49,914,498 | S_#8 | 7.28^E^-07 | 0.18 | 8.6^E^-03 | 0.34 | C |
| S | 20 | 2,246,105 | S_#9 | 4.07^E^-06 | 0.15 | 1.9^E^-02 | 0.17 | C |
| S | 20 | 39,076,484 | S_#10 | 9.13^E^-07 | 0.18 | 9.7^E^-03 | 0.20 | C/M/F |

NA = Proportion de la variance phénotypique non déterminée (NA).

**Table S3:** The t-test results. Degree of significance (p-value) of the phenotypic contrast (difference between means) for Ca, K, P and S content between lines contrasting for the allele carried at each QTL (of the eight co-identified) in three environments. The three trials were conducted in Ottawa (ON) in 2017 (17) and 2018 (18), with (I) or without (N) supplemental irrigation. Phenotypic contrasts were declared significant using a Bonferroni correction ( α = 0.05/n, where n is the total number of the QTLs for a trait) significant differences are indicated using an asterisk.

| **Traits** | **Gm** | QTLs | Environments | | |
| --- | --- | --- | --- | --- | --- |
|  |  |  | I17 | I18 | N18 |
| Ca | 06 | Ca_#3 | 1.3E-02* | 3.6E-03** | 2.3E-02* |
|  | 09 | Ca_#4 | 7.3E-01ns | 7.9E-01ns | 3.6E-01ns |
| K | 04 | K_#1 | 2.4E-04*** | 6.5E-03** | 2.3E-05*** |
|  | 10 | K_#3 | 1.7E-02* | 1.4E-01ns | 1.7E-04*** |
| P | 04 | P_#1 | 3.2E-04*** | 3.5E-03** | 2.6E-04*** |
| S | 10 | S_#4 | 3.4E-04*** | 8.5E-03** | 5.6E-04*** |
|  | 15 | S_#7 | 1.3E-04*** | 7.0E-02ns | 4.2E-05*** |
|  | 20 | S_#10 | 4.6E-03** | 7.5E-03** | 4.2E-04*** |

ns: not significant, *p*-value > 0.025, ** and *** Significant, *p*-value ≤ 0.001 and ≤ 0.0001, respectively.

**Table S4:** Summary of localized GWAS results showing the new peak SNPs (in bold) and old peak SNP associated with seeds Ca, K, P and S content.

| **Trait** | **Gm** | **QTL N°** | **Peak SNP** | **P** | **R^2^** | **FDR** | **Effect** | **MODELS** |
| --- | --- | --- | --- | --- | --- | --- | --- | --- |
| Ca | 06 | Ca_#3 | **3,487,809** | **1.11E-08** | **0.23** | **2.4E-03** | **-0.07** | C/M |
|  |  |  | 3,354,869 | 2.94E-08 | 0.20 | 4.5E-03 | -0.06 |  |
| K | 04 | K_#1 | **49,072,328** | **1.64E-06** | **0.18** | **3.7E-03** | **-0.30** | C/F |
|  |  |  | 49,071,552 | 1.75E-06 | 0.17 | 6.1E-03 | -0,30 |  |
|  | 10 | K_#3 | **1,614,105** | **1,59E-10** | **0.32** | **3.3E-05** | **-0.57** | C/M/F |
|  |  |  | 1,925,709 | 4.31E-10 | 0.31 | 4.9E-05 | -0,57 |  |
| P | 04 | P_#1 | **49,072,328** | **3.51E-08** | **0.22** | **7.4E-03** | **0.30** | C/M/F |
|  |  |  | 49,071,286 | 6.12E-08 | 0.22 | 1.5E-02 | -0.30 |  |
| S | 10 | S_#4 | **1,609,531** | **1.24E-08** | **0.23** | **1.2E-04** | **-0.46** | C/M |
|  |  |  | 1,602,998 | 2.84E-08 | 0.23 | 4.0E-03 | -0.46 |  |
|  | 20 | S_#10 | **39,064,722** | **1.44E-07** | **0.18** | **1.9E-02** | **0.23** | C/M/F |
|  |  |  | 39,076,484 | 9.13E-07 | 0.18 | 9.7E-03 | 0.20 |  |
