# Supplementary material for: Identification of loci controlling mineral element concentration in soybean seeds
Source: BMC Plant Biol. 2020 Sep 7;20:419. doi: 10.1186/s12870-020-02631-w (PMC7487956; doi:10.1186/s12870-020-02631-w)
Supplement: Supplementary file 2 — Additional file 2 Fig. S1: Distribution of the 2.18 M SNPs in the soybean genome. Fig. S2: Quantile-quantile (Q-Q) plot of p-values from the association study. Fig. S3: The boxplot and p-values for t-test result. Fig. S4: Identification of candidate genes underlying QTLs. Fig. S5: Candidate gene haplotypes and their phenotypic contrast. [file 12870_2020_2631_MOESM2_ESM.docx]

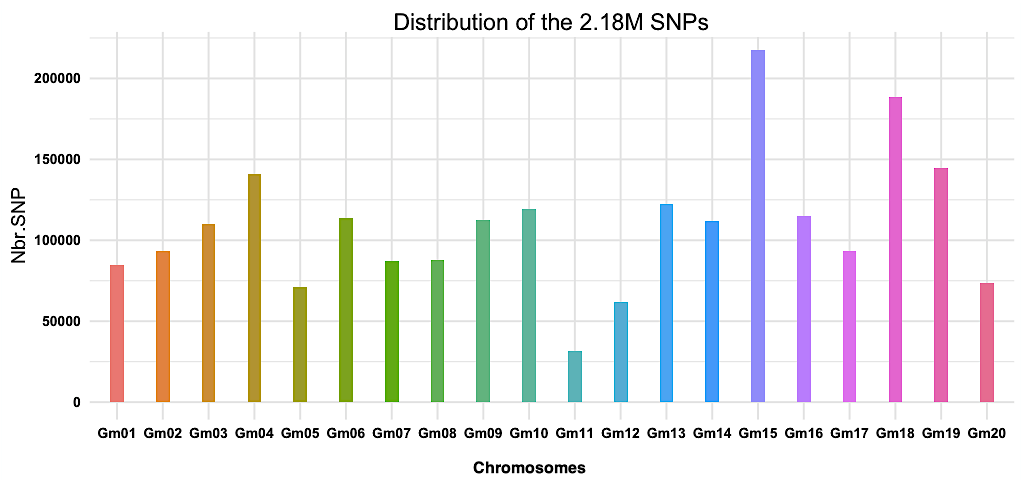


Figure S1: Distribution of the 2.18M SNPs in the soybean genome

**
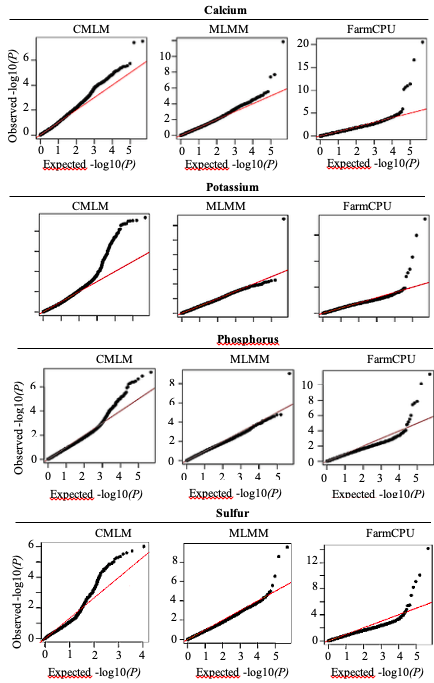
**

Figure S2: Quantile-quantile (Q-Q) plots for the four elements studied (Ca, K, P and S) using CMLM, MLMM and FarmCPU. The Y-axis is the observed negative base 10 logarithm of the *p*-values, and the X-axis is the expected observed negative base 10 logarithm of the *p*-values under the assumption that the *p*-values follow a uniform (0,1) distribution.


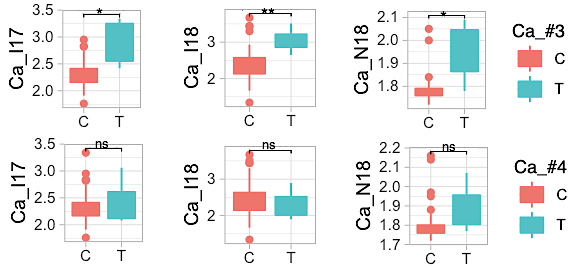


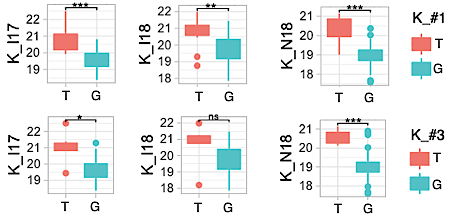


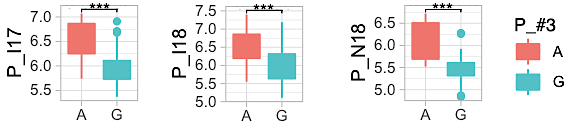


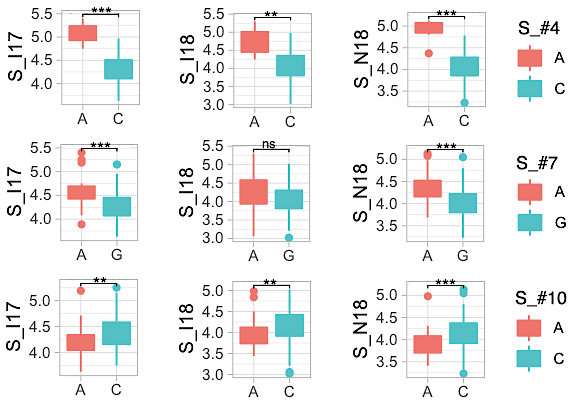


Figure S3: The means differences between allelic classes of the reported QTLs in validation population. The three trials were conducted in Ottawa (ON) in 2017 (17) and 2018 (18), with (I) or without (N) supplemental irrigation. Phenotypic contrasts were declared significant using a Bonferroni adjusted correction (α = 0.05/n where n is the total number of the QTLs for a trait) and significant differences are indicated using an asterisk. ns: not significant, *p*-value > 0.025, ** and *** Significant, *p*-value ≤ 0.001 and ≤ 0.0001, respectively.


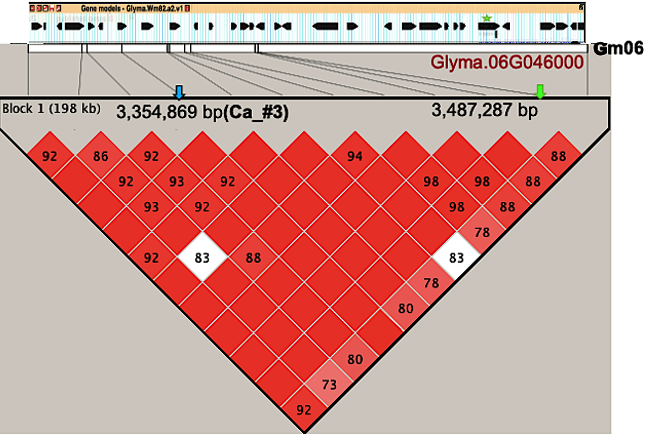


Figure S4a: Genic landscape in haplotype block containing the peak SNP (QTL, Ca_#3). Top panel: position and orientation of 30 gene models present in the 199-kb region that is defined by the left-most (Gm06:3,321,336) and right-most (Gm06:3,520,462) markers that are in perfect LD with the peak SNP (Gm06:3,354,869). The most likely candidate gene (Glyma.06G046000, Calcium ion transport) is highlighted with a green asterisk. Bottom panel: pairwise LD among markers falling within the defined genomic region of interest. LD is indicated as D’x100 and the empty red squares indicate complete LD (D’=1). The position of the peak SNP (blue arrow) and candidate gene (green arrow) are shown.


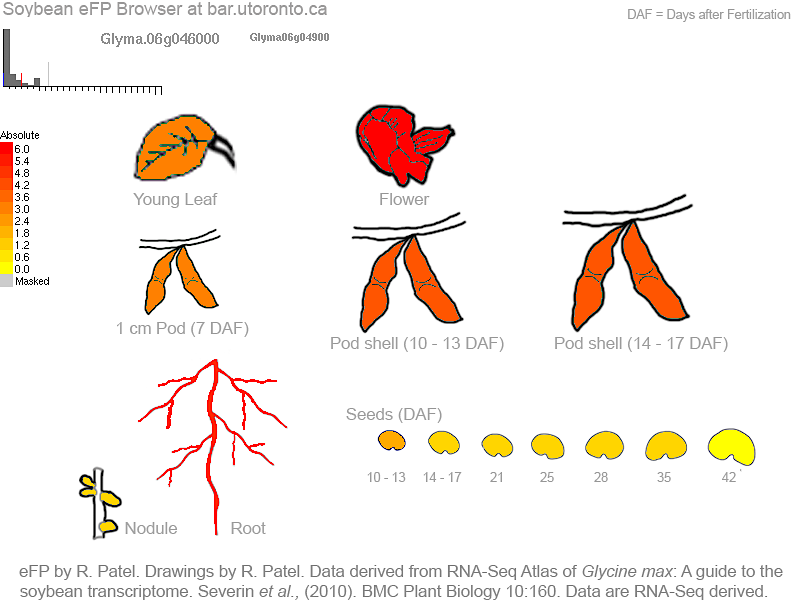


Figure S4b: Gene expression profile in soybeans tissues. Expression strength coded by color: yellow = low, red = high. As shown, Glyma.06G046000 is expressed in root, flowers, pod shell and seeds. Data derived from RNA-seq of Glycine max, published by (Severin et al. 2010) form eFP browser view ([www.bar.utoronto.ca](http://www.bar.utoronto.ca)).


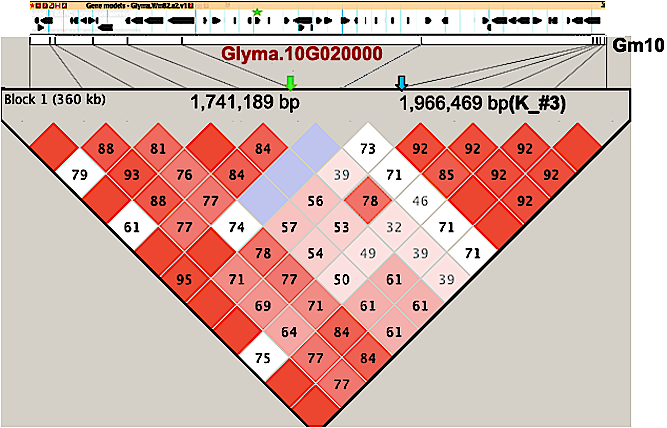


Figure S4c: Genic landscape in haplotype block containing the peak SNP (QTL, K_#3). Top panel: position and orientation of 43 genes models present in the 360-kb region that is defined by the left-most (Gm10:1,611,678) and right-most (Gm10:1,972,235) markers that are in perfect LD with the peak SNP (Gm06:1,966,469). The most likely candidate gene (Glyma.10G020000, Potassium ion transport) is highlighted with a green asterisk. Bottom panel: pairwise LD among markers falling within the defined genomic region of interest. LD is indicated as D’x100 and the empty red squares indicate complete LD (D’=1) while purple empty square indicate no LD (D’= 0). The position of the peak SNP (blue arrow) and candidate gene (green arrow) are shown.


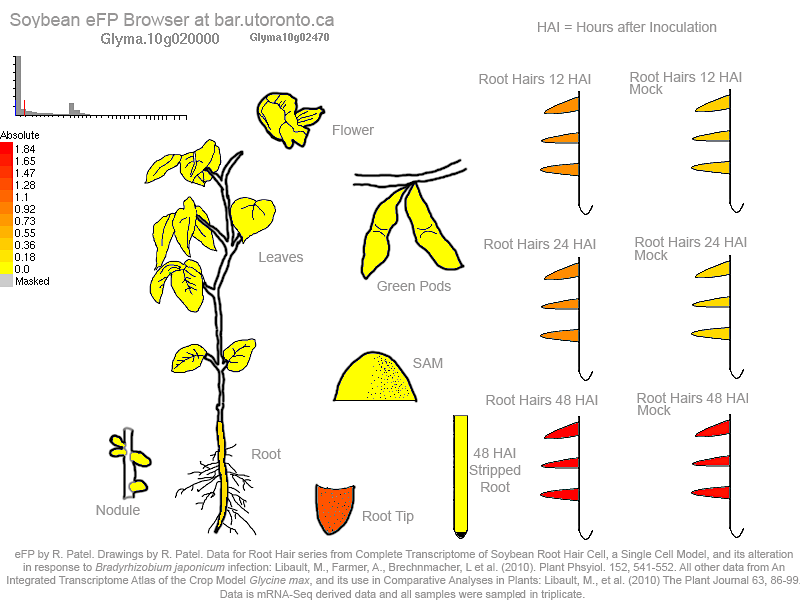


Figure S4d: Gene expression profile in soybeans tissues. Expression strength coded by color: yellow = low, red = high. As shown, Glyma.06G046000 is expressed in root tips and hairs. Data derived from RNA-seq of *Glycine max*, published by Libault et al. (2010) form eFP browser view ([www.bar.utoronto.ca](http://www.bar.utoronto.ca)).

.


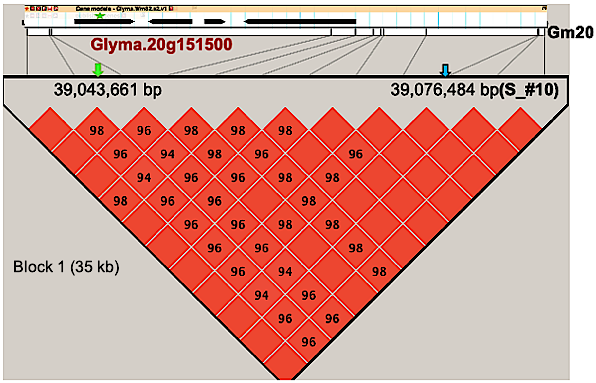


Figure S4e : Genic landscape in haplotype block containing the peak SNP (QTL,S_#10). Top panel shows the position and orientation of all four genes models present in the QTL region 35-kb region that is defined by the left-most (Gm20: 39,042,071) and right-most (Gm20: 39,076,880) markers that are in perfect LD with the peak SNP (Gm20:39,076,484). The most likely candidate gene (Glyma.20G151500, Sulfate assimilation) is highlighted with a green asterisk. Bottom panel: pairwise LD among markers falling within the defined genomic region of interest. LD is indicated as D’x100 and the empty squares indicate complete LD (D’=1). The position of the peak SNP (blue arrow) and candidate gene (green arrow) are shown.


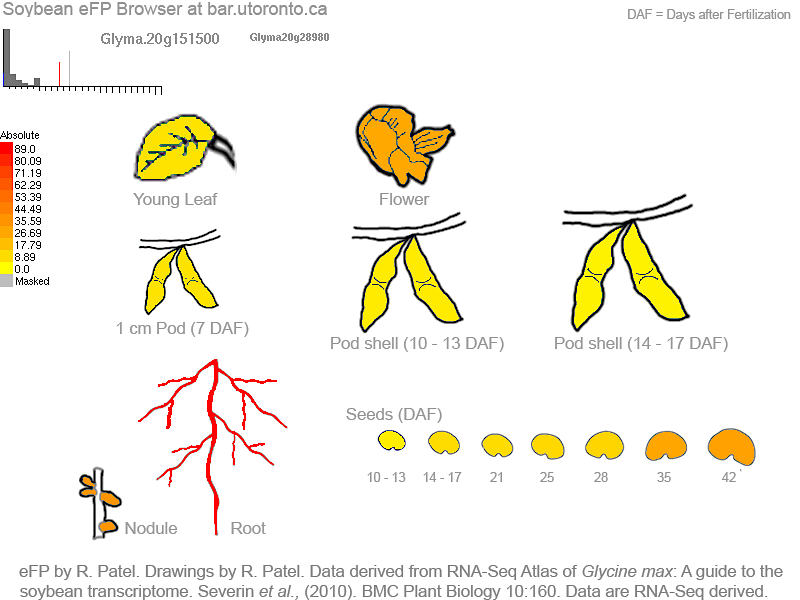


Figure S4f : Gene expression profile in soybeans tissues. Expression strength coded by color: yellow = low, red = high. As shown, Glyma.20G0151500 is most highly expressed in roots . Data derived from RNA-seq of Glycine max, published by (Severin et al. 2010) form eFP browser view ([www.bar.utoronto.ca](http://www.bar.utoronto.ca)).


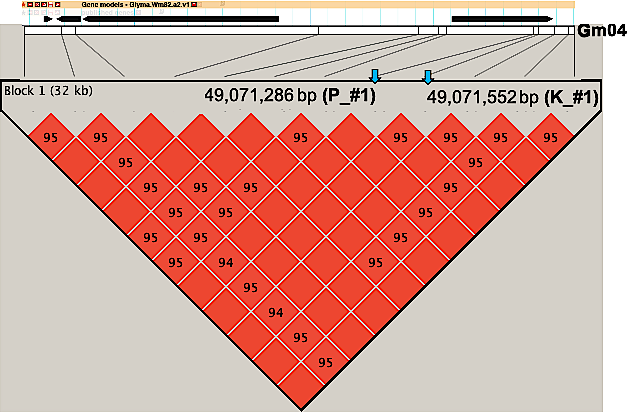


Figure S4g : Genic landscape in haplotype block containing the peak SNP (QTL, P_#1 and K_#1). Top panel: position and orientation of 4 genes models present in the 32-kb region that is defined by the left-most (Gm04: 49,041,258) and right-most (Gm04: 49,073,656) markers that are in perfect LD with the peaks SNPs (Gm04:49,071,286 and Gm04: 49,071,552). No candidate gene falling within the defined LD blocks and meeting our criteria was found. Bottom panel: pairwise LD among markers falling within the defined genomic region of interest. LD is indicated as D’x100 and the empty squares indicate complete LD (D’=1). The position of the peaks SNPs (blue arrow) are shown.


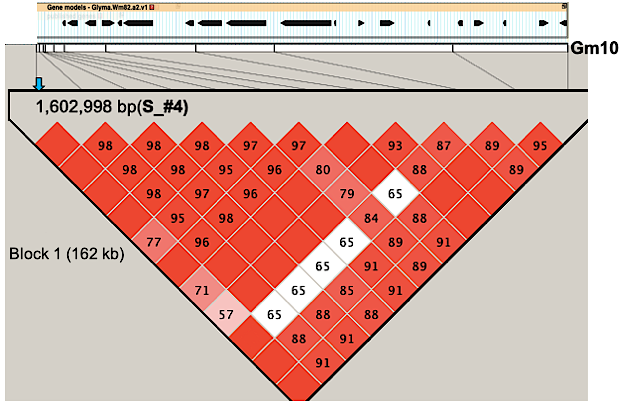


Figure S4h : Genic landscape in haplotype block containing the peak SNP (QTL, S_#4). Top panel: position and orientation of 18 genes models present in the 162-kb region that is defined by the left-most (Gm10: 1,602,998) and right-most (Gm10: 1,764,775) markers that are in perfect LD with the peaks SNPs (Gm10: 1,602,998). No candidate gene falling within the defined LD blocks and meeting our criteria was found. Bottom panel: pairwise LD among markers falling within the defined genomic region of interest. LD is indicated as D’x100 and the empty squares indicate complete LD (D’=1). The position of the peak SNP (blue arrow) is shown.


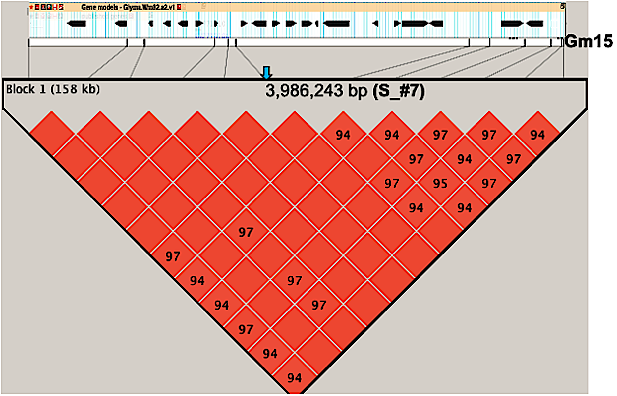


Figure S4i : Genic landscape in haplotype block containing the peak SNP (QTL, S_#7). Top panel: position and orientation of 20 genes models present in the 158-kb region that is defined by the left-most (Gm15: 3,924,891) and right-most (Gm15: 4,082,937) markers that are in perfect LD with the peaks SNPs (Gm15: 3,986,243). No candidate gene falling within the defined LD blocks and meeting our criteria was found. Bottom panel: pairwise LD among markers falling within the defined genomic region of interest. LD is indicated as D’x100 and the empty squares indicate complete LD (D’=1). The position of the peak SNP (blue arrow) is shown.

1. (b)


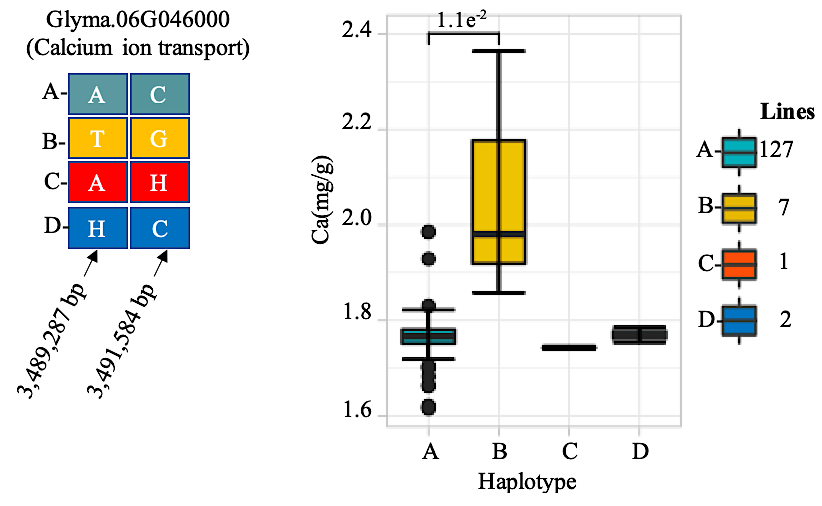


1. (b)


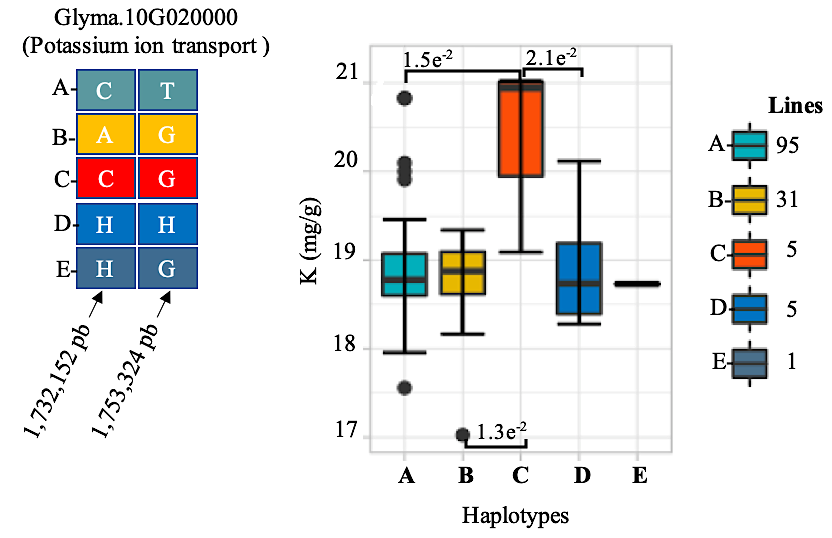


1. (b)


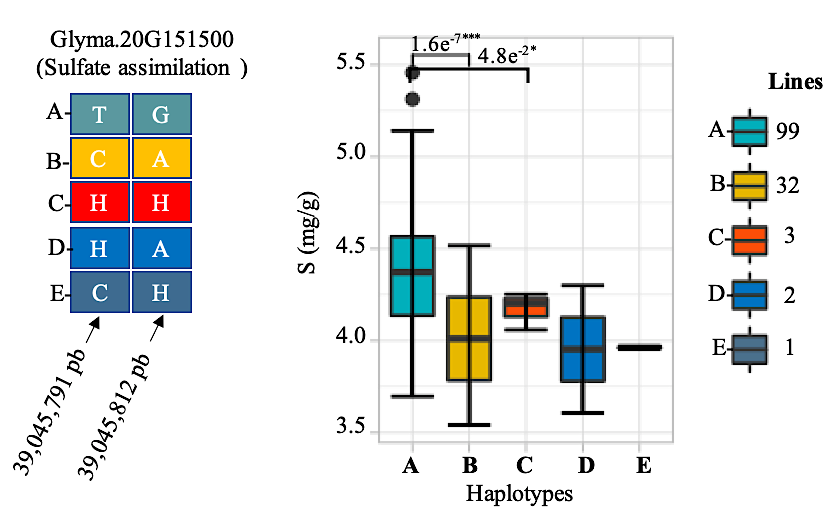


Figure S5: (a) SNPs that capture the haplotype within candidate gene and (b) a t-test (difference between means) between haplotypes of candidate genes Glyma.10G020000, Glyma.10G020000 and Glyma.20G151500, respectively involved in calcium, potassium ion transports and sulfate assimilation in soybean. Only significant differences are shown (*p*-value ≤ 0.05), otherwise means are not significantly different.
